# Comparison of Pathway Analysis Approaches Using Lung Cancer GWAS Data Sets

**DOI:** 10.1371/journal.pone.0031816

**Published:** 2012-02-21

**Authors:** Gordon Fehringer, Geoffrey Liu, Laurent Briollais, Paul Brennan, Christopher I. Amos, Margaret R. Spitz, Heike Bickeböller, H. Erich Wichmann, Angela Risch, Rayjean J. Hung

**Affiliations:** 1 Prosserman Centre for Health Research, Samuel Lunenfeld Research Institute, Mount Sinai Hospital, Toronto, Ontario, Canada; 2 Department of Medicine and Medical Biophysics, Ontario Cancer Institute/Princess Margaret Hospital, Toronto, Ontario, Canada; 3 International Agency for Research on Cancer (IARC), Lyon, France; 4 Department of Epidemiology, The University of Texas M. D. Anderson Cancer Center, Houston, Texas, United States of America; 5 Department of Genetic Epidemiology, University Medical Center, University of Goettingen, Goettingen, Germany; 6 Institute of Epidemiology I, Helmholtz Center Munich, Neuherberg, Germany; 7 Division of Epigenomics and Cancer Risk Factors, German Cancer Research Center, Heidelberg, Germany; Vanderbilt University Medical Center, United States of America

## Abstract

Pathway analysis has been proposed as a complement to single SNP analyses in GWAS. This study compared pathway analysis methods using two lung cancer GWAS data sets based on four studies: one a combined data set from Central Europe and Toronto (CETO); the other a combined data set from Germany and MD Anderson (GRMD). We searched the literature for pathway analysis methods that were widely used, representative of other methods, and had available software for performing analysis. We selected the programs EASE, which uses a modified Fishers Exact calculation to test for pathway associations, GenGen (a version of Gene Set Enrichment Analysis (GSEA)), which uses a Kolmogorov-Smirnov-like running sum statistic as the test statistic, and SLAT, which uses a p-value combination approach. We also included a modified version of the SUMSTAT method (mSUMSTAT), which tests for association by averaging χ^2^ statistics from genotype association tests. There were nearly 18000 genes available for analysis, following mapping of more than 300,000 SNPs from each data set. These were mapped to 421 GO level 4 gene sets for pathway analysis. Among the methods designed to be robust to biases related to gene size and pathway SNP correlation (GenGen, mSUMSTAT and SLAT), the mSUMSTAT approach identified the most significant pathways (8 in CETO and 1 in GRMD). This included a highly plausible association for the acetylcholine receptor activity pathway in both CETO (FDR≤0.001) and GRMD (FDR = 0.009), although two strong association signals at a single gene cluster (*CHRNA3-CHRNA5-CHRNB4*) drive this result, complicating its interpretation. Few other replicated associations were found using any of these methods. Difficulty in replicating associations hindered our comparison, but results suggest mSUMSTAT has advantages over the other approaches, and may be a useful pathway analysis tool to use alongside other methods such as the commonly used GSEA (GenGen) approach.

## Introduction

Genome wide association studies (GWAS) examine the association of hundreds of thousands of genetic variants with disease or other phenotypes. These studies have successfully identified associations between genetic variants and outcome, such as associations between SNPs at the 15q25 and 5p region and lung cancer risk [1], [2], [3], [4], [5], [6]. GWAS of lung cancer and other diseases generally identify only a few SNPs that are associated with disease and these usually have small effect sizes. For instance, the per allele odds ratio for variants which implicate acetylcholine receptor genes at 15q25 with lung cancer risk is about 1.3 [1], [2], [5]. SNPs with weaker effects could be missed given the stringent requirements needed for adjustment for multiple comparisons.

Pathway analysis has been proposed as a complementary approach to single SNP analyses in GWAS. Pathway analysis groups genes that are related biologically and tests whether these gene groups are associated with outcome. Although outcome associated with variation at many genes may be too small to detect in GWAS using single SNP analysis, associations may be detected from the joint effect of many weaker signals at genes grouped into a pathway based on shared biological function. Other benefits of this approach are the substantial reduction of the multiple testing burden once genes are grouped into pathways for association testing [7] and the incorporation of biological knowledge into the analysis, which is not accounted for in GWAS.

The number of methods developed for pathway analysis continues to increase. Many on-line programs offer a simple gene set enrichment approach that uses some form of Fisher's Exact test to determine over-representation of genes within a pathway. Generally, a gene is assigned a P-value (usually obtained from the SNP most strongly associated with outcome at a gene) and an arbitrary cut-off (e.g., P≤0.05) is used to separate genes strongly associated with outcome from other genes. A Fishers Exact calculation is then used to test for within pathway enrichment of genes strongly associated with outcome. This approach does not account for linkage disequilibrium patterns among SNPs at different genes in the pathway. As well, it may over-estimate the significance of pathways with large genes (i.e., many SNPs), since selecting the most significant SNP when there are many SNPs at a single gene is more likely to find a strong association between gene and outcome by chance [8], [9].

The popular GSEA approach generally uses the SNP most strongly associated with outcome at each gene to represent gene-outcome associations. Some implementations take into account linkage disequilibrium among SNPs and gene size bias by performing phenotype (case-control status) permutations and using normalization routines. Genes are first ranked by size of their test statistic for association with outcome. A Kolmogorov-Smirnov-like running sum statistic is then used to test for enrichment of highly ranked genes within pathways, by comparing the pathway test statistic to its null distribution as determined by the phenotype permutations [9], [10]. Other approaches, for example the SUMSTAT approach which uses the sum of χ^2^ statistics assigned to genes as a pathway test statistic [11], can be adapted to use phenotype permutations and normalization methods. Alternatives to these gene set enrichment approaches, such as methods of combining P-values (similar to meta-analyses), have also been proposed for pathway analysis. Some of these, incorporate methodology that accounts for potential bias related to gene size or correlation among SNPs [12], [13].

We compare four pathway analysis methods. These include a simple gene enrichment approach in EASE, which calculates a modified Fishers Exact probability [14], GSEA (using the GenGen program) [9], [10], a modified SUMSTAT approach, and SLAT, a P-value combination approach [12]. The first method is representative of early simpler approaches which use the Fishers Exact test, while the others, as outlined above, are more sophisticated and designed to address biases related to gene size and linkage disequilibrium among SNPs. We compare and contrast the results from analyses using these methods in two lung cancer GWAS data sets.

## Materials and Methods

### Samples

Data were used from case-control GWAS of lung cancer risk. These included lung cancer cases and controls from Central Europe [2], Toronto [2] and Germany (HGF study) [15], [16] and non-small cell lung cancer cases and controls from Texas (MD Anderson Cancer Centre) [1]. Genotyping was performed using either the Illumina HumanHap300 or HumanHap550 chips. Data from the four studies were combined into two data sets: 1) Central Europe and Toronto (CETO); and 2) Germany and Texas (GRMD), in order to reach adequate sample size and statistical power to detect associations in the pathway analyses. The choice of which data sets to combine was predominantly made to ensure similar sample sizes in the two independent analyses. Table 1 provides further details related to these studies.

**Table 1:** Comparison of study designs, selected epidemiologic variables, genotyping platforms and results.

							Combined Data Sets		
Study	Type	Case/control	Sex (Males)	Median Age (range)	Ever smokers	CHIP/SNP number	SNPs mapping to genes	# of genes after SNP mapping	# of GO Pathways
1^st^ Stage									
Central Europe	Cases: hospital Controls: hospital and population	1926/2522	75%	61 (25–89)	Cases: 93% Controls 65%	Illumina HumanHap300 (317,139)			
Toronto	Cases: hospital Controls: clinic and population	333/506	42%	58 (20–85)	Cases: 71% Controls: 56%	Illumina HumanHap300 (317,139)			
Central Europe/Toronto combined	Cases: hospital Controls: various	2258/3027†	70%	60 (20–89)	Cases: 89% Controls: 63%	Illumina HumanHap300 305,326†	161,435	17811	421
2^nd^ Stage									
Texas (MD Anderson)	Cases: hospital Controls: clinic	1154/1137	57%	62 (31–92)	Cases: 100% Controls: 100%	Illumina HumanHap300 317,498			
Germany (DKFZ/LUCY/KORA)	Cases: hospital Controls: population	504/484	57%	46 (27–51)	Cases: 93% Controls: 54%	Illumina HumanHap550 561,466			
Texas Germany combined	Cases: hospital Controls: various	1639/1618†	57%	57 (27–92)	Cases: 98% Controls: 86%	Illumina HumanHap300 302,334†	160,726	17805	420

† After implementing data quality measures described in methods.

### Selection of pathway analysis methods

Pathway analysis methods were identified through literature review. Methods implemented in the programs EASE [14], GenGen (developed from GSEA) [9], [10], and SLAT [12] were chosen because they were widely used and/or representative of other pathway analysis approaches. We chose the SUMSTAT method based on a report indicating it had superior power to detect pathway associations than GSEA or Fishers Exact methods [11]. For this method an in-house SAS program was developed. The methods are described here briefly, with details provided in the original publications.

### Description of gene set analysis methods

With the exception of SLAT, pathway analysis methods described here require assignment of a test statistic (or P-value) to each gene representing its association with outcome. We used the common practice of assigning each gene the most significant test statistic from all SNP associations tests for the gene [8], [9].

Input for EASE requires that genes significantly associated with outcome are distinguished from all other genes, using a pre-specified cut-off (e.g., P≤0.05). Enrichment for significant genes in each pathway is then tested using the EASE score, a modified Fishers Exact probability representing the upper bound of jackknife Fisher exact probabilities. Global FDRs are calculated to account for multiple comparisons [14].

GenGen is adapted from Gene Set Enrichment Analysis (GSEA), used originally for microarray analysis [17]. Genes are ranked in descending order according to size of the initial association statistic. A weighted Kolmogorov-Smirnov-like running sum statistic is then calculated that reflects over representation of higher ranked genes in a pathway in the gene list. The weight takes on the values of SNP test statistics representing genes in the list. A normalized enrichment statistic (NER) is calculated for observed data, followed by phenotype permutations which give permuted NER values, creating the null distribution from which pathway association P-values are determined. FDRs are used to account for multiple comparisons [9].

The modified SUMSTAT (mSUMSTAT) approach, that we developed, is adapted from Tintle et al. [11]. The approach is similar to that used in GenGen but the pathway test statistic is calculated by averaging χ^2^ test statistics within each pathway. The equation below shows the calculation of the normalized mean value of the observed χ^2^ statistic, where S refers to a specific gene set and π denotes the permutation. The normalized permuted statistic is calculated the same way.
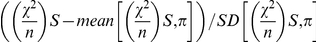



The p-value is determined by comparing the normalized mean value of the χ^2^ statistic to the normalized permuted mean χ^2^ statistics [18] and a FDR is calculated according to Wang et al. [9]. This method contrasts to that of Tintle et al., [11] through the calculation of a normalized test statistic, and use of phenotype permutations instead of randomly selected gene sets to determine the null distribution.

The SLAT program calculates P-values for association of SNPs with outcome for a defined pathway (as in this study), gene, or region. P-values reaching a specific threshold are combined into a test statistic. The statistic is calculated for observed and phenotype permuted data which permits determination of a pathway P-value [12]. No particular method for adjusting for multiple comparisons is provided by the authors. (We used the Benjamini-Hochberg correction to calculate FDRs for this method).

### Analysis details

SNPs were excluded when the P-value for HWE in controls was ≤0.001 (consistent with previous pathway analysis studies [9], [11]), the minor allele frequency was <1%, and genotype was missing in >5% of individuals. In addition, SNPs from the HumanHap550 chip that were used in the German GWAS were excluded if there was no corresponding SNP from MD Anderson (the study with which German GWAS data was combined).Subjects with sex discrepancies (based on heterozygosity rate at chromosome X) and those with >10% missing SNPs were excluded.

Unconditional logistic regression, using PLINK 1.05 [19] generated allelic χ^2^ values for SNPs for each data set, CETO and GRMD, for use in the programs EASE, GenGen and mSUMSTAT. Permuted SNP association results were generated for GenGen and mSUMSTAT using 1000 logistic regression runs with case-control status randomly shuffled for each run. Logistic regression analyses were adjusted for sex, age and country of origin. The SLAT program performed its own SNP association tests for its pathway analysis, which does not include adjustment for covariates.

SNPs were assigned to a gene if they were within 20 kb of the gene. A SNP to gene linking file and GO level 4 pathway database file, both obtained from the GenGen web site, were used to link SNPs, genes and pathways. Only pathways with 15 to 200 genes were included to avoid testing overly large or small GO pathways [6]. The χ^2^ of the most significant SNP at gene was assigned to that gene. This χ^2^ statistic was used to assign the cut-off value of P≤0.05 to identify strongly associated genes for analysis with EASE. The same χ^2^ statistic was used in the calculation of the pathway test statistics for GenGen and mSUMSTAT. All SNPs at each gene were used as input for the calculation of pathway P-values for SLAT.

The influence of gene size on pathway ranking of the four pathway analysis methods was investigated using linear regression analysis (SAS 9.2: SAS Institute Inc., Cary, North Carolina). Median gene size (median number of SNPs per gene) was calculated for each top pathway and included as the outcome variable in a model with pathway analysis method (treated as a categorical variable and coded into four dummy variables) as the main effect and number of genes per pathway included as a potential confounder.

## Results

Table 2 shows the number of significant pathways identified by the four pathway analysis methods in CETO and GRMD using a FDR of ≤0.05 as the criterion to determine statistical significance. EASE identified 10 pathways as associated with lung cancer risk in the two data sets, 7 in CETO, 5 in GRMD, with two significant pathways common to both data sets. The mSUMSTAT method identified 8 pathways as significant, 8 in CETO, 1 in GRMD with one being common to both data sets. SLAT identified five pathways as significant, three in GRMD and two in CETO.

**Table 2:** Number of significant pathway associations (using FDR< = 0.05) for Central Europe-Toronto (CETO) and Germany-MD Anderson (GRMD) by pathway analysis method.

Data set	EASE	GenGen	mSUMSTAT	SLAT
CETO	7	0	8	2
GRMD	5	0	1	3
Both CETO and GRMD	2	0	1	0
Total	10	0	8	5

Since EASE identified 10 significant pathways, more than the other methods, Table 3 shows the top 10 pathways identified in CETO and GRMD by all pathway analysis methods (taken from lists comprising results from both data sets). An FDR of ≤0.05 in both data sets was used as the criteria for a replicated result. Transmission of nerve impulse and the Ras guanyl nucleotide exchange factors pathways were identified by EASE as associated with lung cancer in CETO and GRMD (Table 3). The acetylcholine receptor activity pathway was identified as associated with lung cancer in CETO and GRMD by mSUMSTAT. This pathway contains the *CHRNA3-CHRNA5-CHRNB4* gene cluster at 15q25, where GWAS have identified several SNPs associated with lung cancer risk [1], [2], [5]. This pathway was the highest ranked pathway in CETO using the GenGen method (FDR = 0.19) (Table 3). In GRMD, this pathway was ranked 16^th^ among all pathways (not shown) by GenGen. The FDR was 0.43, but it was accompanied by a nominally significant P-value (P = 0.004). Other significant pathway associations in CETO had corresponding nominally significant P-values in GRMD, specifically: heme metabolic process, porphyrin metabolic process, pigment biosynthetic process, and 4 iron, 4 sulfur cluster binding using mSUMSTAT; and low-density lipoprotein binding using EASE. SLAT identified regulation of cell migration as significantly associated with lung cancer in GRMD, with a corresponding nominally significant P-value in CETO (Table 3).

**Table 3:** Comparison of FDRs (top line) and P-values (in brackets) for Central Europe-Toronto (CETO) and Germany-MD Anderson (GRMD) for top lung cancer risk associated pathways identified by different analysis methods using GO level 4 pathways.

EASE			GenGen			mSUMSTAT			SLAT		
Go	FDR (P-value)		Go	FDR (P-value)		Go	FDR (P-value)		Go	P-value	
Pathway	CETO	GRMD	pathway	CETO	GRMD	pathway	CETO	GRMD	pathway	CETO	GRMD
**nerve impulse**	**<0.001 (<0.001)**	**<0.001 (0.005)**	acetylcholine receptor †	0.194 (0.001)	0.430 (0.004)	**acetylcholine receptor** †	**<0.001 (<0.001)**	**0.009 (<0.001)**	***regulation of cell migration***	***0.0048***	***0.0002*** ‡
**Ras-GEF** †	**<0.001 (0.007)**	**<0.001 (<0.001)**	immune response†	1.00 (0.735)	0.271 (0.002)	***heme metabolic***	***0.003 (<0.001)***	***0.245 (0.005)***	growth factor activity	0.2727	<0.0001‡
***LDL binding*** †	***<0.001 (0.005)***	***0.400 (0.021)***	chloride ion binding	0.316 (0.002)	0.707 (0.213)	***porphyrin metabolic***	***0.010 (<0.001)***	***0.329 (0.029)***	gland development	0.6215	0.0003‡
chloride ion binding	<0.001 (0.003)	1.00 (0.065)	interleukin-2 biosynthetic	1.00 (0.446)	0.294 (0.002)	***pigment biosynthetic***	***0.033 (<0.001)***	***0.290 (0.043)***	glycoprotein metabolic	<0.0001‡	0.1223
interleukin-2 biosynthetic	1.00 (0.419)	<0.001 (<0.001)	cytokine metabolic	1.00 (0.278)	0.314 (0.003)	***4 iron, 4 sulfur cluster binding***	***0.042 (0.001)***	***0.334 (0.011)***	chromatin assembly	0.0002‡	0.7325
carboxylic acid transport	<0.001 (0.005)	0.955 (0.498)	heme metabolic	0.381 (0.001)	0.506 (0.056)	chromatin assembly	0.036 (0.001)	0.813 (0.669)	complement activation †	0.0015	0.2004
regulation of cell migration	1.00 (0.456)	<0.001 (0.006)	complement activation †	0.361 (0.004)	0.819 (0.567)	complement activation †	0.034 (0.003)	0.737 (0.536)	response to steroid hormone	0.0014	0.0418
sensory organ development	1.00 (0.487)	<0.001 0.002)	somatic recombination†	1.00 (0.308)	0.358 (0.002)	antigen processing†	0.032 (0.003)	0.570 (0.280)	regulation of axonogenesis	0.0383	0.0014
phospholipid transporter	<0.001 (<0.001)	1.00 (0.280)	peptide receptor†	1.00 (0.453)	0.370 (0.009)	mRNA binding	0.052 (0.001)	0.316 (0.051)	retrograde transport, GER†	0.0007	0.4841
muscle contraction	<0.001 (<0.001)	0.394 (0.085)	positive reg Phosphorous†	1.00 (0.639)	0.380 (0.018)	anion transport	0.055 (<0.001)	0.735 (0.557)	fatty acid regulation	0.0009	0.4935

Bold: significant after adjustment for multiple comparisons (FDR≤0.05) in both CETO and GRMD.

Bold with italics: significant after adjustment for multiple comparisons in one data set (FDR≤0.05), nominal significance in other (P≤0.05).

Underline: Top pathways identified by more than one pathway analysis method within a data set.

† Abbreviated GO category name. Full category names as follows: Ras-GEF: Ras guanyl-nucleotide exchange factor; LDL binding: low-density lipoprotein binding; acetylcholine receptor: acetylcholine receptor activity; immune response: adaptive immune response based on somatic recombination of immune receptors built from immunoglobulin superfamily domains; complement activation: complement activation, classical pathway; somatic recombination: somatic recombination of immunoglobulin gene segments; peptide receptor: G-protein coupled peptide receptor activity; positive reg phosphorous: positive regulation of phosphorus metabolic process; antigen processing: antigen processing and presentation of peptide antigen via MHC Class I; retrograde transport, GER: Retrograde vesicle mediated transport, golgi to endoplasmic reticulum.

‡ Significant based on Benjamini-Hochberg FDR calculation.

Other than the acetylcholine receptor activity pathway, which was identified by both mSUMSTAT and GenGen as a top pathway, there were few top pathways identified by more than one method. Chloride ion binding was associated with risk in CETO according to EASE and GenGen. Complement activation-classical pathway was associated with lung cancer risk in CETO according to GenGen, mSUMSTAT and SLAT. Heme metabolic process was identified as associated with risk in CETO by GenGen and mSUMSTAT. Chromatin assembly was associated with lung cancer risk in CETO according to mSUMSTAT and SLAT. Interleukin-2 biosynthetic process was identified as associated with risk by EASE and GenGen in GRMD. Regulation of cell migration was associated with risk for GRMD according to EASE and SLAT (Table 3). Anion transport was identified as a top pathway by mSUMSTAT but 35 of 102 genes in this pathway were included in the chloride ion binding pathway (64 genes), identified as a top pathway by EASE and GenGEN (gene number in pathways calculated following SNP mapping). Likewise, 16 of 18 genes in the interleukin 2 pathway (EASE) are included among the 65 genes in the cytokine metabolic pathway (GenGen). Other top pathways identified by different methods shared genes but the overlap was 12% or less based on shared genes for the larger of the two pathways (e.g., 20 of 50 positive regulation of phosphorous pathway genes (GenGen) are included in the growth factor metabolism pathway (SLAT), which has 165 genes).

The EASE method selected pathways with greater gene size (defined using the median number of SNPs per gene) than the other methods. The average gene size for the top EASE pathways shown in Table 3 was 12.2 SNPs per gene, whereas average top pathway gene size was 8.4 for GenGen, 7.4 for mSUMSTAT, and 8.7 for SLAT. Regression analysis, where pathway analysis method was coded into four dummy variables, produced a statistically significant association between the EASE method and gene size (P = 0.02).

As two methods identified acetylcholine receptor activity as a top pathway we examined this association in more detail. SNPs near the *CHRNA3*-*CHRNA5*-*CHRNB4* gene cluster showing strong associations with lung cancer risk, are in strong LD, and there is overlap among SNP test statistics assigned to these genes (i.e., the test statistic for the same SNP was assigned to both *CHRNA5* and *CHRNA3*). These pathway characteristics may bias pathway association signals [20], [21] To evaluate whether the pathway analysis was driven by a single associated gene or the gene cluster, we examined the effect of removing the *CHRNA5* gene (where the putative causal variant is located) and the entire gene cluster from analyses using mSUMSTAT and GenGen. Removing *CHRNA5* had no influence on mSUMSTAT results in CETO (*CHRNA5*: P< = 0.001, FDR≤0.001) but FDRs fell well below the 0.05 significance level in GRMD (*CHRNA5*: P = 0.002, FDR = 0.37). Removing *CHRNA5* from the GenGen analysis resulted in reduced strength of association in CETO (P = 0.003, FDR< = 0.48) but virtually no change in GRMD (P = 0.01, FDR< = 0.41). However, removal of the entire gene cluster resulted in marked reduction of the FDR and loss of significance in the two data sets for both pathway analysis methods (mSUMSTAT without CHRNA3-CHRNA5-CHRNB4: CETO: P = 0.19, FDR = 0.56 GRMD: P = 0.71, FDR = 0.82; GenGen without CHRNA3-CHRNA5-CHRNB4 CETO: P = 0.11, FDR = 1.00 GRMD: P = 0.32, FDR = 0.76).

**Figure 1:**
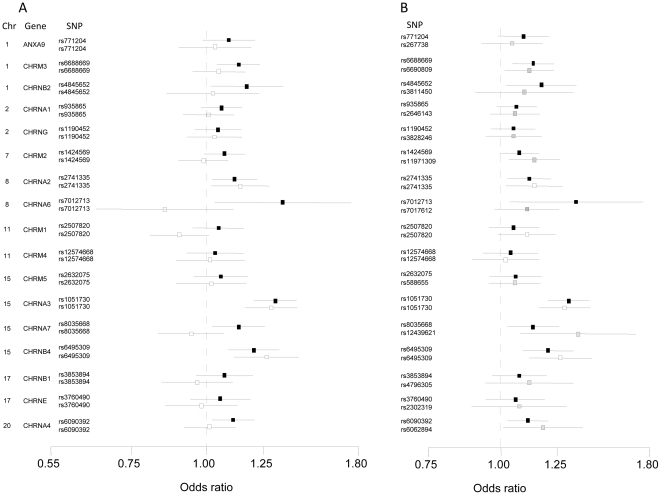
Comparison of odds ratios for acetylcholine receptor pathway showing. A) the most significant SNP for each gene used in Central Europe-Toronto analysis and odds ratios for same SNPs for Germany MD Anderson); B) the most significant SNP assigned to each gene in either data set (i.e., the actual SNPs used in pathway analyses in the two data sets). Chromosome number (Chr) and genes for both graphs are shown on left. (Central Europe – Toronto SNPs: solid fill, Germany MD Anderson matching SNPs: no fill; Germany MD Anderson top SNP (differing from Central Europe-Toronto): grey fill). A) Reference allele same in both Central Europe-Toronto and Germany-MD Anderson but chosen to show positive association for Central Europe-Toronto. B) Reference allele always chosen to show positive association. *CHRNA5* is excluded as SNPs are identical to those representing *CHRNA3*. Odds ratios adjusted for age, sex and country of study.

We further explored the association of this pathway with risk by graphing odds ratios and 95% confidence limits for acetylcholine receptor pathway SNPs and genes produced by unconditional logistic regression analyses. Figure 1A shows odds ratios for specific SNPs assigned to genes (i.e., the most significant SNP for each gene) for the CETO analysis and for comparison, odds ratios for these same SNPs for GRMD. In addition to SNPs in the *CHRNA3-CHRNA5-CHRNB4* gene cluster, a SNP at *CHRNA2* showed a nominally significant association with risk in both data sets (CETO: P = 0.012; GRMD: P = 0.022). Figure 1B shows odds ratios for the most significant SNP assigned to each gene in either data set (i.e., the actual SNPs used in pathway analyses in the two data sets). Additional nominally significant associations were found for *CHRM3* (CETO: P = 0.003; GRMD: P = 0.028), *CHRNA7* (CETO: P = 0.016; GRMD: P = 0.009), and *CHRNA4* (CETO: P = 0.012; GRMD: P = 0.038) in both data sets. In total, 6 of 8 genes associated with risk in CETO were associated with risk in GRMD, a result greater than expected by chance given the number of SNPs at each gene.

## Discussion

Four pathway analysis methods were compared by using each to test association of GO level 4 pathways with lung cancer risk in two lung cancer GWAS data sets. Methods compared included four gene set enrichment approaches, EASE, GenGen, mSUMSTAT and a p-value combination approach, SLAT. After adjustment for multiple comparisons using an FDR of less than or equal to 0.05 as the criterion for a significant association, EASE and mSUMSTAT identified more pathways associated with lung cancer risk across the two datasets (10 and 8 respectively) than did GenGen (no pathways), or SLAT (5 pathways). EASE and mSUMSTAT also identified pathways that were significantly associated with risk in both data sets: transmission of nerve impulse and Ras guanyl nucleotide exchange factor by EASE; and the acetylcholine receptor activity pathway by mSUMSTAT. There was limited agreement among the different methods in the identification of top ranked pathways. Comparing genes among top pathways chosen by each method showed only a modest degree of overlap.

In comparing pathway analysis methods, we examined whether the number of SNPs per gene in pathways influenced the selection of top pathways. The results indicated EASE, identified top pathways with a significantly greater median number of SNPs per gene than the other methods. This result is not unexpected. For all gene set enrichment methods we used the common approach of assigning the most significant SNP to represent each gene. Genes with more SNPs, generally large genes, are more likely to be assigned a SNP with a high association statistic, which can lead to over estimation of significance of pathways with large genes (gene size bias) [8], [9]. We acknowledge that large genes might be more likely to harbour multiple variants which are truly associated with outcome, but our comments focus on statistical properties of the methods, specifically the potential for false positives resulting from gene size bias. EASE, which uses a relatively simple approach based on the Fishers Exact test, is susceptible to this bias. Normalization routines and phenotype permutations incorporated into GenGen and mSUMSTAT protect against this bias [6], [22]. SLAT is also protected against this bias as it uses all SNPs in a pathway for analysis and incorporates a phenotype shuffling routine [12]. The more robust design of GenGen, mSUMSTAT and SLAT provides an additional benefit, as these methods account for correlation among SNPs within pathways.

A critical aspect of this comparison was the use of replication of top pathways across CETO and GRMD to help evaluate the relative performance of these methods. However, based on an FDR of ≤0.05, few replicated associations were found. Lack of study power may in part account for the small number of replicated associations. In particular GRMD (cases = 1639, controls = 1618) may have had insufficient sample size to detect associations found in CETO (cases = 2258, controls = 3027). Heterogeneity between data sets might also have contributed to small number of replicated associations, as the German sample was restricted to subjects under age 50, and the MD Anderson GWAS included only ever smokers. Therefore, GRMD subjects were younger and had a higher proportion of ever smokers compared to CETO subjects.

Among the three methods (GenGen, mSUMSTAT and SLAT) that are robust against gene size bias only mSUMSTAT identified a replicated association. This was for the acetylcholine receptor activity pathway. The association of this pathway with risk is not unexpected as several SNPs at or near the *CHRNA3*-*CHRNA5*-*CHRNB4* gene cluster are associated with both lung cancer risk [1], [2], [5] and nicotine addiction [5], [23], [24]. It is of interest that the GenGen method also identified acetylcholine receptor activity as the top ranked pathway in CETO and one of the most highly ranked pathways in GRMD, although the result was not significant in either data set after correcting for multiple comparisons using the FDR. We note that the associations found for this pathway was driven by the *CHRNA3*-*CHRNA5*-*CHRNB4* gene cluster, as demonstrated by the dramatic reduction of strength of association (according to the FDR) found for both the mSUMSTAT and GenGen methods when data were reanalyzed with these three genes removed from the pathway. This may complicate the interpretation of the observed association as ideally, significant pathways should not be identified from a signal that might ultimately represent a single gene or variant [20], [21] We point out, however, that there are two independent risk associated loci in this region [25] and it is currently not clear which genes in the region are causally related to disease risk. It is preferable then that pathways such as these are identified to be associated with outcome by the analysis method, and the researcher can then follow-up with additional exploratory analyses. Further investigation of this pathway did suggest that allowing the same SNP to represent both *CHRNA5* and *CHRNA3* in the analysis overestimated significance in the GRMD data set for mSUMSTAT and the CETO data set for Gengen. Results from analyses that excluded *CHRNA5* are likely the most appropriate for this pathway.

For the purpose of further comparing pathway associations across data sets we used a less restrictive criterion for a replicated pathway association (a significant FDR in one data set and a nominally significant association (P< = 0.05) in the second). This permitted additional associations to be identified, although with less confidence than those identified using the original criterion. The mSUMSTAT method found four potential risk associated pathways with a significant FDR in CETO and nominally significant P-values in GRMD: heme metabolic process, porphyrin metabolic process, pigment biosynthesis and 4 iron, 4 sulfur cluster binding. The heme metabolic and porphyrin metabolic pathways show a high degree of overlap. All four of these pathways include *IREB2* which is in the same region of strong LD that includes the *CHRNA3*-*CHRNA5*-*CHRNB4* cluster. SLAT identified one pathway, regulation of cell migration, using this same criterion.

Overall, our results (along with insights from other comparisons discussed below) suggest mSUMSTAT should be considered when choosing a method for pathway analysis. Lack of strong replication of pathway associations makes it difficult to evaluate GenGen and SLAT against one another. However, the GenGen approach appears to have some advantages. GenGen results provided some support for an association of the acetylcholine receptor pathway with risk, and like mSUMSTAT this method allows for the incorporation of covariates, whereas the SLAT program does not have this capability. Finally, GenGen is commonly used and has provided other plausible associations in pathway analyses of GWAS data sets [10]. On the other hand, the utility of SLAT is difficult to assess given our results and further evaluation of this method is needed. The rest of the discussion focuses on mSUMSTAT and GenGen.

Our mSUMSTAT method contrasts to that of Tintle et al. [11] through calculation of a normalized test statistic, and use of phenotype permutations instead of randomly selected gene sets to determine the null distribution. These changes were introduced to address gene size bias and maintain the correlation structure among SNPs in a pathway.

Some simulation results suggest that approaches that use the sum or average of the χ^2^ as a pathway test statistic will be more powerful than those that use the weighted Kolmogorov-Smirnov-like running sum statistic incorporated into GenGen and related GSEA approaches. Tintle et al. found that the original SUMSTAT test statistic was more powerful than a GSEA approach in a comparison where random gene sets were used to construct the null distribution for both methods [11]. Efron and Tibshirani found generally lower p-values using mean test statistics when compared to GSEA in simulated gene expression analyses [18].Their analysis used a t-test instead of a χ^2^ statistic, allowing for gene expression comparisons of two groups. Permutation and normalization approaches were the same as used here, except normalization for GSEA also incorporated means and standard deviations calculated from permutations with random gene sets. Our results are consistent with these studies in that mSUMSTAT identified several significant associations in CETO and GRMD (with one of these replicated in both data sets), while GenGen did not, suggesting that mSUMSTAT may have greater power to detect associations.

Since the strongest association found by GenGen and mSUMSTAT was for the acetylcholine receptor pathway we graphed odds ratios and confidence limits to further explore the pathway association. Despite weak association signals found for these regions when the *CHRNA3*-*CHRNA5*-*CHRNB4* cluster was removed from analyses, the graphical presentation of results suggests that SNPs outside of this gene cluster may contribute to the association, as suggested by replicated associations across the two data sets. This association appeared more convincing when comparing the most significant SNPs representing each gene across the two data sets (gene based comparison) as opposed to comparing the most significant SNPs at each gene in CETO to the same SNPs in GRMD (variant based comparison). Better evidence for replication could result from a gene based approach versus a SNP based approach if multiple SNPs capture the causal variant(s) more completely than single SNPs for some pathway genes. This can be advantageous to pathway analysis approaches which can rely on gene based association signals to better replicate pathway associations.

In summary, this study compared several different pathway analysis approaches in two lung cancer GWAS data sets comprising four studies. Difficulties in replicating associations across studies hindered our comparison and we cannot clearly establish one pathway analysis method as superior to the others. However, the mSUMSTAT approach did demonstrate several strengths such as a highly plausible association with the acetylcholine receptor pathway and several additional suggestive associations, while accounting for correlation among SNPs and gene size bias. Since different pathway analysis methods can produce different results using the same data set (as was seen here), it is best to use more than one method when examining pathway associations with disease risk [26]. We suggest that the mSUMSTAT method could be used in combination with other methods, such as the better known GenGen approach, in pathway analysis investigations.
